# Yeast Frataxin Is Stabilized by Low Salt Concentrations: Cold Denaturation Disentangles Ionic Strength Effects from Specific Interactions

**DOI:** 10.1371/journal.pone.0095801

**Published:** 2014-05-06

**Authors:** Domenico Sanfelice, Rita Puglisi, Stephen R. Martin, Lorenzo Di Bari, Annalisa Pastore, Piero Andrea Temussi

**Affiliations:** 1 MRC National Institute for Medical Research, The Ridgeway, London, United Kingdom; 2 Dipartimento di Chimica e Chimica Industriale, Pisa, Italy; 3 Scuola Normale Superiore, Pisa, Italy; 4 Dipartimento di Chimica, Università di Napoli Federico II, Napoli, Italy; Consiglio Nazionale delle Ricerche, Italy

## Abstract

Frataxins are a family of metal binding proteins associated with the human Friedreich's ataxia disease. Here, we have addressed the effect of non-specifically binding salts on the stability of the yeast ortholog Yfh1. This protein is a sensitive model since its stability is strongly dependent on the environment, in particular on ionic strength. Yfh1 also offers the unique advantage that its cold denaturation can be observed above the freezing point of water, thus allowing the facile construction of the whole protein stability curve and hence the measurement of accurate thermodynamic parameters for unfolding. We systematically measured the effect of several cations and, as a control, of different anions. We show that, while strongly susceptible to ionic strength, as it would be in the cellular environment, Yfh1 stability is sensitive not only to divalent cations, which bind specifically, but also to monovalent cations. We pinpoint the structural bases of the stability and hypothesize that the destabilization induced by an unusual cluster of negatively charged residues favours the entrance of water molecules into the hydrophobic core, consistent with the generally accepted mechanism of cold denaturation.

## Introduction

Understanding the factors governing the thermal stability of proteins and correlating them with their sequence and structure is a complex and multifaceted problem that can nevertheless provide important information on the molecular forces involved in protein folding.

Frataxins are a family of essential acidic proteins with an α/β fold that is highly conserved both in sequence and structure from bacteria to humans [1]. They play a crucial role in mitochondrial iron metabolism as shown by the fact that neuronal death observed in Friedreich's ataxia (FRDA, OMIM:229300) patients arises from disregulation of iron homeostasis, with concomitant oxidative damage [1], [2]. In agreement with a role in iron metabolism, frataxins bind both Fe(II) and Fe(III) [3], [4] but with unexpected if not unique features, which make them unusual proteins. The interaction is purely electrostatic, in agreement with the conserved acidic pIs of frataxins, and involves semi-conserved aspartates and glutamates in helix 1. Accordingly, the interaction is not restricted to a limited number of cations but a large negatively charged surface is provides multiple sites of interaction. In addition to Fe^2+^ and Fe^3^, it is known that other divalent and trivalent cations (Mg^2+^, Ca^2+^, Zn^2+^, Co^2+^, Al^3+^ and various lanthanides) [5] bind to the same sites but with lower affinity and specificity.

In spite of their high degree of conservation, the mid point of the thermal unfolding (T_m_) of different frataxin orthologs are very different [6]. Of the proteins studied so far, the human and *Escherichia coli* proteins are stable, having melting points around 50–60°C, while frataxins from the psychrophilic Antartic bacterium *Psychromonas ingrahamii* and *Saccharomices cerevisiae* have melting points around 30°C [7]. These differences are even more marked if one considers that yeast Yfh1 is also one of the few proteins whose cold denaturation can be observed at temperatures above the freezing point of water and at physiological pH values, without the need for the addition destabilizing agents [3]. Furthermore, whereas the nature of the intrinsic instability of the protein from a psychrophilic organism makes functional sense and could be related to adaptation to low temperatures, less obvious are the bases of the behavioral differences of proteins from different mesophilic organisms.

An interesting observation is the relationship between stability and ion concentrations. While a specific effect of divalent cations is expected, since frataxins are iron binding proteins, there seems to also be a strong non-specific dependence on ionic strength. The effect is most evident for the least stable proteins for which the increases in *T_m_* values are as large as 10–15°C, even for modest increases of ionic strength [6]. Cold denaturation of Yfh1 is observable only when the protein is completely depleted of salt as even small concentrations of NaCl can cause significant stabilization. Only highly deionized water should therefore be used for thermodynamic studies of this protein (S. Gianni, personal communication).

Prompted by these unusual observations and in the attempt to identify the molecular mechanisms of protein stability and cold denaturation, we became interested in the relationship between specific and non-specific ion binding and stabilization effects. The ease of measuring cold denaturation and hence the whole stability curve makes Yfh1 a system uniquely suited to measuring the influence of external agents on thermal stability. The influence of external agents, such as salts, on the thermal stability of proteins is generally measured by monitoring changes in *T_m_*, the midpoint transition temperature for unfolding. However, as shown in a study on the influence of alcohols, even a large stabilization may only produce a relatively small change in *T_m_* [8]. The best way to measure protein stability is to construct the whole stability curve by determining the ΔG for unfolding as a function of temperature. This is a difficult task for most proteins because only when physical measurements cover, at least in part, both unfolding transitions is it possible to construct the whole curve of thermal stability, as defined by Becktel and Schellman [9].

Here, we report a spectroscopic study on the stability of Yfh1 in aqueous solutions containing varying concentrations of different salts. We examined the influence of salts on the stability of frataxins exploiting the possibility of measuring the stability of Yfh1 more accurately and, through a systematic study, to untangle the relative influence of cations and anions in several of the salts previously employed. We first studied the effect of cations by measuring the influence of NaCl, KCl, MgCl_2_ and CaCl_2_ by using circular dichroism (CD) and then extended the study to several anions, i.e. the sodium salts of F^−^, Cl^−^, H_2_PO_4_^−^ and SO_4_^2−^. We chose to examine monovalent and divalent cations preceding iron in the periodic table to distinguish specific binding effects from the influence of the bulk environment. These findings were confirmed and substantiated by nuclear magnetic resonance spectroscopy (NMR). We can now pinpoint from our studies the residues that are specifically involved in destabilization and indicate the forces that contribute to destabilization of the fold. These findings are also useful in attempting to unravel the structural bases of the cold denaturation phenomenon and to understand more the properties of frataxins.

## Materials and Methods

### Sample preparation

Both unenriched and ^15^N-enriched Yfh1 were expressed in E. *coli* as described by He et al. [4]. Bacteria were grown in minimal medium using ammonium chloride as the sole source of nitrogen. An EDTA tablet was added before cell lysis. Yfh1 purification involved two precipitation steps with (NH_4_)_2_SO_4_, dialysis and then anion exchange chromatography with a GE Healthcare Q-sepharose column using a NaCl gradient. After a dialysis, the protein was further purified by a chromatography with a GE Healthcare Phenyl Sepharose column with a decreasing gradient of (NH_4_)_2_SO_4_. Purity of the recombinant proteins was checked by SDS-PAGE after each step of the purification.

### Far-UV CD measurements

Far-UV CD spectra were recorded on a Jasco J-710 spectropolarimeter. Samples were prepared using a Yfh1 concentration of 10 µM in 10 mM HEPES buffer at pH 7.5 and with a variety of salts: NaCl (Sigma-Adrich), KCl (Aldrich), MgCl_2_ (Aldrich), CaCl_2_ (J.T.Baker), NaF (May&Baker), NaH_2_PO_4_ (Carlo Erba), Na_2_SO_4_ (Sigma-Aldrich) and NaI (Carlo Erba). Baseline correction was performed by subtraction of the appropriate buffer spectrum. Thermal unfolding curves were obtained by monitoring the ellipticity at 220 nm using a Jasco J-815 CD spectropolarimeter equipped with a Jasco CDF-4265/15 Peltier unit. Measurements were repeated at least twice on independent protein preparations to ensure reproducibility of the results. All samples used Yfh1 at 10 µM in a 10 mM HEPES buffer at pH 7.5 and varying concentrations of NaCl, KCl (Fisher Scientific), MgCl_2_, CaCl_2_, FeSO_4_, NaF (Sigma-Aldrich), NaH_2_PO_4_ (Acros Organics) and Na_2_SO_4_ (Merck). 2 mm path length cuvettes (Hellma) were used with a heating rate of 2°C/min in the temperature range 0–80°C. In the case of the ferrous salt rigorous anaerobic conditions were achieved by preparing the samples in a glove box, sealing the cuvette and performing the measurements under N_2_ (g) flow.

### NMR spectroscopy

NMR spectra were acquired on a Bruker AVANCE spectrometer operating at 600 MHz ^1^H frequency. Typically, measurements were carried out in a 10 mM HEPES buffer, pH 7.5 using a 0.125 mM uniformly ^15^N-enriched protein. Water suppression was achieved by using WATERGATE [11], HSQC experiments were used as described by Bax et al. [12]. The spectra were processed and zero-filled to the next power of two by using the NMRPipe program [13]. Baseline correction was applied when necessary.

## Results

### Cations versus anions

Circular dichroism (CD) spectroscopy is ideally suited to assess the secondary structure content of proteins under different environmental conditions and consequently measure the relative populations of folded and unfolded species. The thermal stability of Yfh1 in different salts was probed with CD spectroscopy by monitoring the intensity at 220 nm in the temperature range 0–80°C. Many of the studies on the influence of salts on protein stability refer to the so-called Hofmeister series, originally introduced to explain protein solubility [14]. We are aware that the explanation for dependence of protein stability on the original Hofmeister series remains a controversial issue [15]. Notwithstanding, we used this series as a general, albeit coarse, guideline and also because any significant departure from the series can hint at specific interactions.

**Figure 1:**
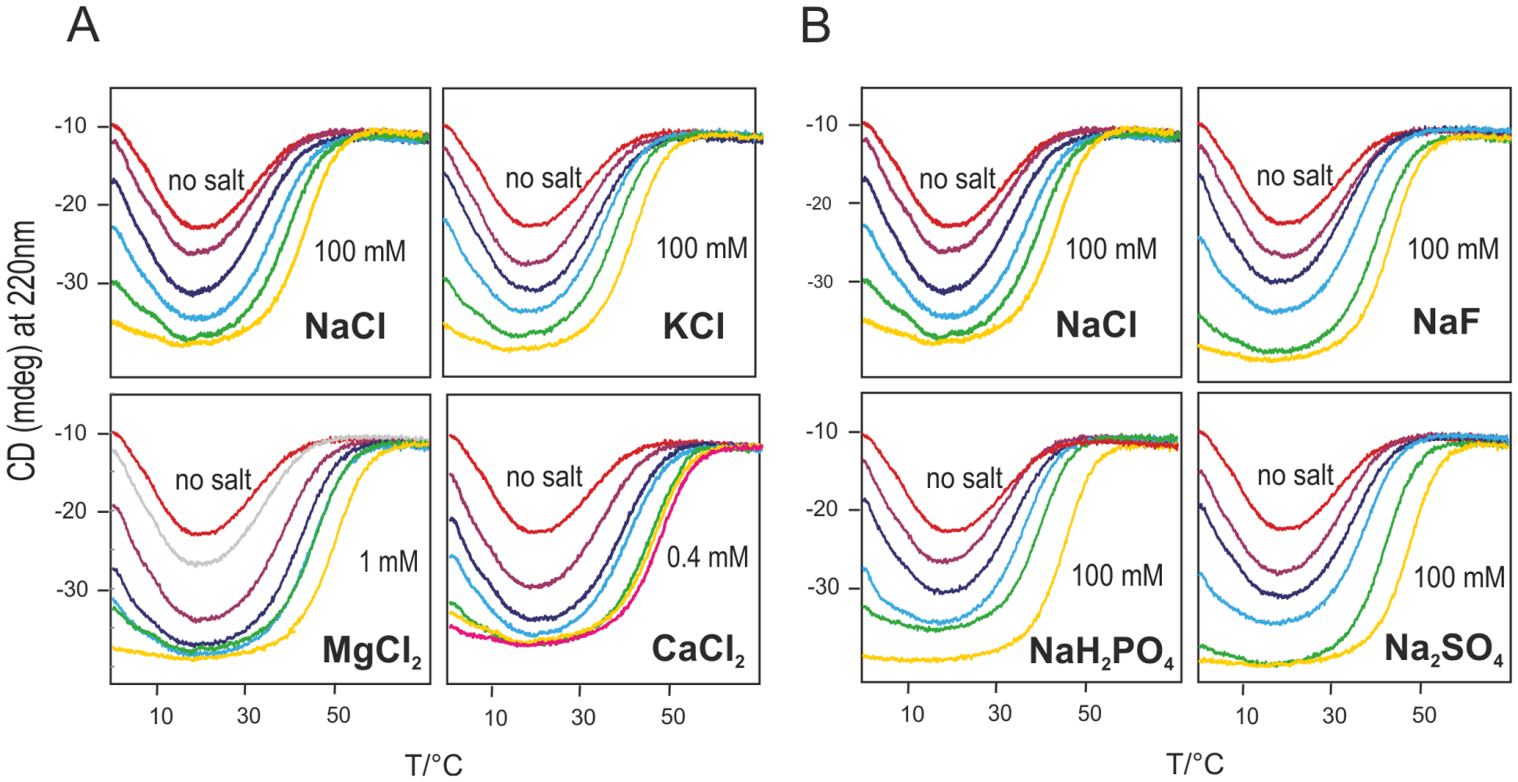
Thermal denaturation curves of Yfh1 in the presence of salts. A) Thermal denaturation curves of 10 µM Yfh1 in a 10 mM HEPES buffer at pH 7.5, measured by monitoring the CD intensity at 220 nm as a function of temperature in the temperature range 0°C to 80°C in the presence of varying concentrations of NaCl, KCl, MgCl_2_ and CaCl_2_. Denaturation curves in the presence of NaCl/KCl shown between the two extremes (“no salt” and 100 mM) have the following concentrations: 5/10/25/50 mM. Denaturation curves in the presence of MgCl_2_ shown between the two extremes (“no salt” and 1 mM) have the following concentrations: 0.05/0.1/0.2/0.3/0.4 mM. Denaturation curves in the presence of CaCl_2_ shown between the two extremes (“no salt” and 0.4 mM) have the following concentrations 0.05/0.08/0.1/0.2/0.3 mM. B) corresponding curves in the presence of varying concentrations of NaF, NaCl, NaH_2_PO_4_ and Na_2_SO_4_. Curves shown between the two extremes (“no salt” and 100 mM) have the following millimolar concentrations: 5/10/25/50.

We selected the chlorides of Na^+^, K^+^, Mg^2+^ and Ca^2+^ for cations and the sodium salts of F^−^, Cl^−^, H_2_PO_4_^−^ and SO_4_^2−^ for anions. To enhance the effect of even very low salt concentrations we chose conditions slightly different from those previously reported [3], i.e. 10 mM HEPES buffer at pH 7.5 instead of 20 mM HEPES buffer at pH 7.0. We first explored the effect of cations. The curves shown in Figure 1A indicate a dramatic increase in the population of the folded species, a corresponding increase of the high temperature melting point (*T_m_*) and a decrease of the cold denaturation temperature (*T_c_*), consistent with a substantial increase in the thermal stability of Yfh1. Our cation series shows a drastic departure from the Hofmeister series: instead of destabilizing the protein, cations are actually very effective in stabilizing Yfh1 but, even more importantly, divalent cations are far more effective than monovalent ones whereas, according to the Hofmeister series, divalent cations are expected to strongly *destabilize* proteins. This finding is consistent with specific binding of to Fe^2+^ and less specific binding of other divalent cations but also shows that any cation can increase stability.

We then explored the effect of four different anions, because it is commonly believed that, in the Hofmeister series, the influence of anions is stronger than that of cations. All the curves (Figure 1B) are consistent with an increase in the thermal stability, albeit in a concentration range much higher than that observed for divalent cations. In principle, reversibility might be affected by the rate of heating. Although a rate of heating of 2°C/min is sufficiently slow, we ran a check with static measurements at a few predefined temperatures, to be sure that the transitions observed in the continuous runs were completely reversible and not influenced by the rate. Figure S1 shows the CD spectra of Yfh1 at 0°C, 20°C and 40°C in the presence of different salts at the same concentration (2 mM).

A difference with respect to the original qualitative investigations [6] is that phosphate, although the most effective of the anions, does not appear to be outstanding, as it is only slightly more effective than, for instance, sulphate.

### Using CD thermograms to detect specific binding

**Figure 2:**
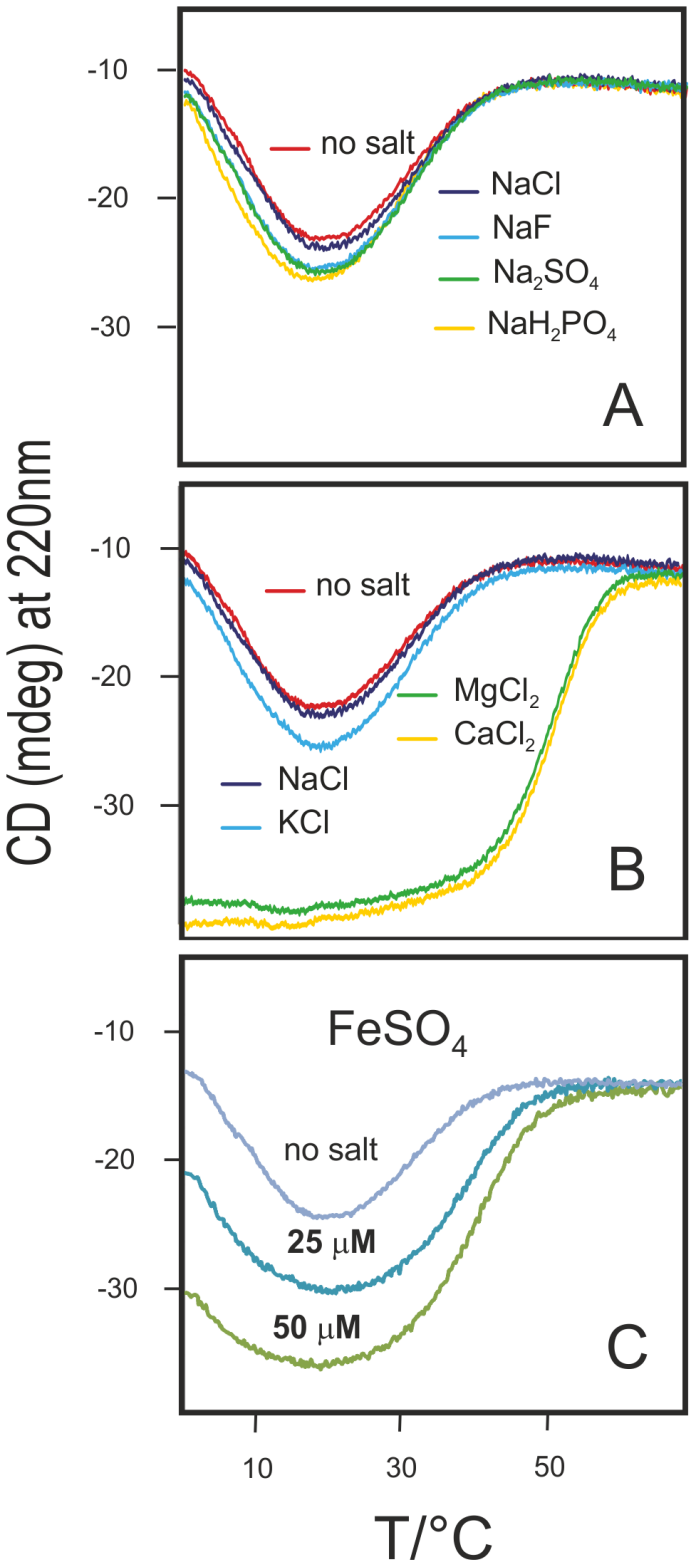
Thermal denaturation of Yfh1 at comparable anion and cation concentrations. Thermal denaturation curves of 10 µM Yfh1 in a 10 mM HEPES buffer at pH 7.5, measured by monitoring the CD intensity at 220 nm as a function of temperature in the temperature range 0°C to 80°C in the presence of A) 2 mM NaF, NaCl, NaH_2_PO_4_ and Na_2_SO_4_ B) 2 mM NaCl, KCl, MgCl_2_ and CaCl_2_, C) 25 and 50 µM FeSO_4_.

An interesting qualitative comparison among different salts can be obtained by plotting thermal denaturation curves for all salts at the same concentration. Figure 2 shows this comparison for a 2 mM concentration (top two panels). A concentration of 2 mM corresponds to a molar ratio (salt:protein) of 200∶1, yet it corresponds only to a modest increase of the CD signal of Yfh1, hinting at a weak unspecific ionic strength effect. Figure 1B shows that a minimum concentration of 5 mM is required to see sizable effects. At concentrations lower than 2 mM, only divalent cations have a sizeable stabilization effect on Yfh1. The stabilization induced by anions at this concentration is so small that it is tempting to attribute it mainly to their counterion (Na^+^).

The difference between monovalent and divalent cations is particularly dramatic. Prompted by the results with Mg^2+^ and Ca^2+^, we also measured the stability of Yfh1 in the presence of Fe^2+^ under rigorous anaerobic conditions. Figure 2C shows the comparison of the thermal denaturation curves of Yfh1 in buffer and in the presence of two very low concentrations of Fe^2+^ (25 and 50 µM). These concentrations correspond roughly to a 2.5∶1 and a 5∶1 ion:protein molar ratio, respectively, and are sufficient to considerably stabilize the protein. Higher concentrations could not be investigated because of protein aggregation and precipitation, but it is clear that iron ions are much more effective than the other divalent cations studied in this work. Accordingly, the action of ferrous ions cannot be attributed solely to a generic interaction with the negatively charged protein surface but rather to specific binding, albeit atypical, iron binding sites.

### Quantifying the effect by thermodynamics parameters

A more quantitative comparison of the influence of different salts on stability can be obtained if the raw CD data are transformed into the corresponding stability curves. This is important because, as previously reported [3], the population of the folded form of Yfh1 in the absence of salts at room temperature is considerably less than 100%. If one assumes a two state equilibrium between folded and unfolded forms, the fraction of folded species at any temperature, f_F_(T), is a function of Δ*G^o^*(T), the Gibbs free energy for unfolding. Since Δ*C_p_* is largely independent of temperature [9], Δ*G* can be expressed by a modified Gibbs-Helmholtz equation in which the reference temperature is the midpoint of the high temperature transition (*T_m_*): 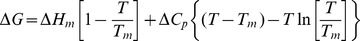

*T_m_*, Δ*H* ^o^(*T_m_*), and Δ*C_p_* can be determined by a nonlinear least squares fit to the observed f_F_(T) values [8], [10]. A detailed description of the procedure for data handling is given in Text S1, where examples of the fits to the thermograms are also shown (Figure S2).

When only the high temperature unfolding can be observed, the fit is rather insensitive to the value of Δ*C_p_*, but yields reliable values when both low and high temperature transitions are observable. All thermodynamic parameters can then be extracted from a plot of Δ*G* ^o^ against temperature, known as the protein stability curve [9]. The errors in Δ*C_p_* and the other parameters are modest because the temperature dependence of Δ*G* is quite sensitive to the value of Δ*C_p_* (which determines the degree of curvature of the stability curve).

Thermodynamic parameters, extracted from the corresponding stability curves, are summarized in Table 1. We calculated thermodynamic parameters only for denaturation curves that showed a significant part of the cold denaturation region because only in these cases does the best fit yield reliable values of Δ*C_p_*. The behaviour of the different salts is qualitatively similar, although the effective concentrations that stabilize the protein, in going from monovalent cations to divalent cations span almost three orders of magnitude. As the salt concentration is increased from the nominal “zero ionic strength” of the HEPES buffer there is a regular increase in all parameters defining stability. The enthalpy difference (Δ*H_m_*), increases from 21 kcal mol^−1^ to a maximum of 46 kcal mol^−1^ for 50 mM NaH_2_PO_4_; *T_m_* goes from 30 °C to almost 45°C for 0.2 mM CaCl_2_; the entropy difference (Δ*S_m_*) increases from 70 cal K^−1^ mol^−1^ to 148 cal K^−1^ mol^−1^ for 50 mM NaH_2_PO_4_ and there is an increase in the difference in free energy at the temperature of the maximum of the stability curve (Δ*G_S_*) from 0.24 kcal mol^−1^ to a maximum of 1.97 kcal mol^−1^ for 50 mM NaH_2_PO_4_. The only parameter that decreases regularly as the salt concentration is increased from the nominal “zero ionic strength” is Δ*C_p_*, which goes from 3.0 kcal K^−1^ mol^−1^ in the HEPES buffer to 1.55 kcal K^−1^ mol^−1^ for 0.2 mM CaCl_2_, i.e. it approaches the value of 1.58 kcal K^−1^ mol^−1^ expected for a protein of 111 residues [16]. All data are consistent with the transition from a marginally stable protein to a “*normally*” stable protein as the salt concentration is increased.

**Table 1:** Thermodynamic parameters for thermal unfolding of Yfh1 in the presence of selected salts.

HEPES salt/mM	% folded^a^	ΔH_m_/kcal mol^−1^	T_m_/°C	ΔC_p_/kcal K^−1^ mol^−1^	ΔS_m_/cal K^−1^ mol^−1^	ΔG°^b^_S_/kcal mol^−1^
**HEPES**						
	60±2	21.1±0.1	29.7±0.3	3.04±0.0	70±1	0.24±0.07
**NaF**						
5	63±2	20.1±0	31.4±0.3	2.06±0.02	66±1	0.32±0.05
10	67±2	22.3±0	32.9±0.2	1.95±0.01	72±1	0.40±0.04
25	79±1	29.0±0	35.4±0.1	1.72±0.01	94±01	0.78±0.01
50	93±1	40.0±0	40.3±0.1	1.67±0.01	128±0	1.49±0.01
**NaCl**						
5	62±2	19.4±1	32.0±0.6	2.1±0.02	64±1	0.29±0.08
10	66±2	21.8±1	33.6±0.3	1.93±0.01	71±1	0.39±0.06
25	75±1	26.4±0	35.8±0.2	1.73±0.01	86±1	0.64±0.05
50	86±1	33.0±0	36.6±0.1	1.62±0.01	106±0	1.06±0.03
**NaH_2_PO_4_**						
5	61±2	18.7±0	31.2±0.3	2.80±0.01	61±1	0.27±0.03
10	70±2	20.1±0	33.3±0.2	1.89±0.01	79±1	0.50±0.06
25	88±0.8	36.6±0	36.1±0.1	1.84±0.01	118±1	1.15±0.04
50	97±0.6	46.4±0.1	41.0±0.02	1.7±0.1	148±1	1.97±0.09
**Na_2_SO_4_**						
5	66±4	22.1±0.3	33.8±1	2.01±0.01	72±3	0.39±0.1
10	72±2	25.3±0	35.8±0	1.84±0.01	89±1	0.55±0.06
25	89±1	36.5±0	37.3±0.1	1.75±0.01	117±1	1.02±0.05
50	96±1	45.2±0	42.7±0	1.71±0.01	143±0	1.84±0.02
**KCl**						
5	61±2	18.4±0.1	31.4±1	2.09±0.02	60±1	0.26±0.06
10	64±2	20.1±0.1	32.7±0.4	1.91±0.02	66±1	0.34±0.04
25	73±2	25.4±0	34.2±0	1.76±0.01	83±0	0.59±0.03
50	88±1	34.4±0	36.8±0	1.64±0.01	111±0	1.14±0.01
**MgCl_2_**						
0.05	65±2	21.7±0.1	31.4±0	2.17±0.02	71±1	0.35±0.08
0.1	73±2	25.0±0	34.2±0	1.73±0.01	81±1	0.58±0.06
0.2	88±1	34.4±0	40.7±0	1.61±0.01	110±1	1.14±0.04
0.3	93±1	39.0±0	44.2±1	1.57±0.01	123±0	1.49±0.01
**CaCl_2_**						
0.05	64±2	20.0±0.1	30.9±0	1.92±0.01	66±1	0.35±0.08
0.08	67±2	21.6±0	34.2±0	1.61±0.01	70±1	0.58±0.03
0.1	80±1	27.9±0	36.6±0	1.54±0.01	90±0	1.14±0.02
0.2	93±1	39.6±0	44.7±0	1.55±0.01	125±0	1.55±0.02
**FeSO_4_**						
0.025	76±2	26.7±0	35.0±0	1.71±0.02	87±1	0.66±0.05
0.05	90±1	36.7±0	40.5±0	1.66±0.01	117±0	1.25±0.03

a percentage of folded protein at the temperature of maximum stability.

b ΔG° at the temperature of maximum stability calculated using the Gibbs-Helmholtz equation.

The data of Table 1 allow a facile estimate of relative efficacies of anions and cations in the stabilization of the marginally stable yeast frataxin. We can order the different ions by comparing the concentrations at which Yfh1 attains maximum or near–maximum stability, as measured by the value of Δ*G_S_*, the free energy difference at the temperature corresponding to the maximum of the stability curve. Thus, we may say that NaH_2_PO_4_ appears to be approximately twice as effective as NaCl because a concentration of 25 mM NaH_2_PO_4_ yields a population of 88% folded species with a Δ*G_S_* of 1.15 kcal mol^−1^ vs a population of 86% and a Δ*G_S_* of 1.06 kcal mol^−1^ yielded by a 50 mM solution of NaCl. Using the same criterion, we get the following ordering of the anions: Cl^−^ ≤ F^−^ < SO_4_^2−^ ≤ H_2_PO_4_^−^, which corresponds roughly to what one would expect on the basis of the Hofmeister series. Altogether, as seen from a glance at the curves of Figure 1B, anions require fairly high concentrations to stabilize Yfh1 significantly. The corresponding comparison of cations tells us that the order is: Na^+^ ≤ K^+^ << Mg^2+^ < Ca^2+^ < Fe^2+^. This ordering is the opposite of what is predicted by the Hofmeister series and the difference between divalent and monovalent ions is even more dramatic. Mg^2+^ ions require a concentration of only 0.2 mM to reach a folded population of 88% and a Δ*G_S_* of 1.14 kcal mol^−1^ with respect to the 50 mM Na^+^ that yields a comparable 86% and a Δ*G_S_* of 1.14 kcal mol^−1^; the approximate ratio of efficacies is thus ca. 250 times in favour of Mg^+^. The ratio between Ca^2+^ and Mg^2+^ is ca. 1 and that between Fe^2+^ and Ca^2+^ is ca. 4; so there is a three order of magnitude difference between the concentration required for the most effective divalent ion and that for the monovalent cations.

**Figure 3:**
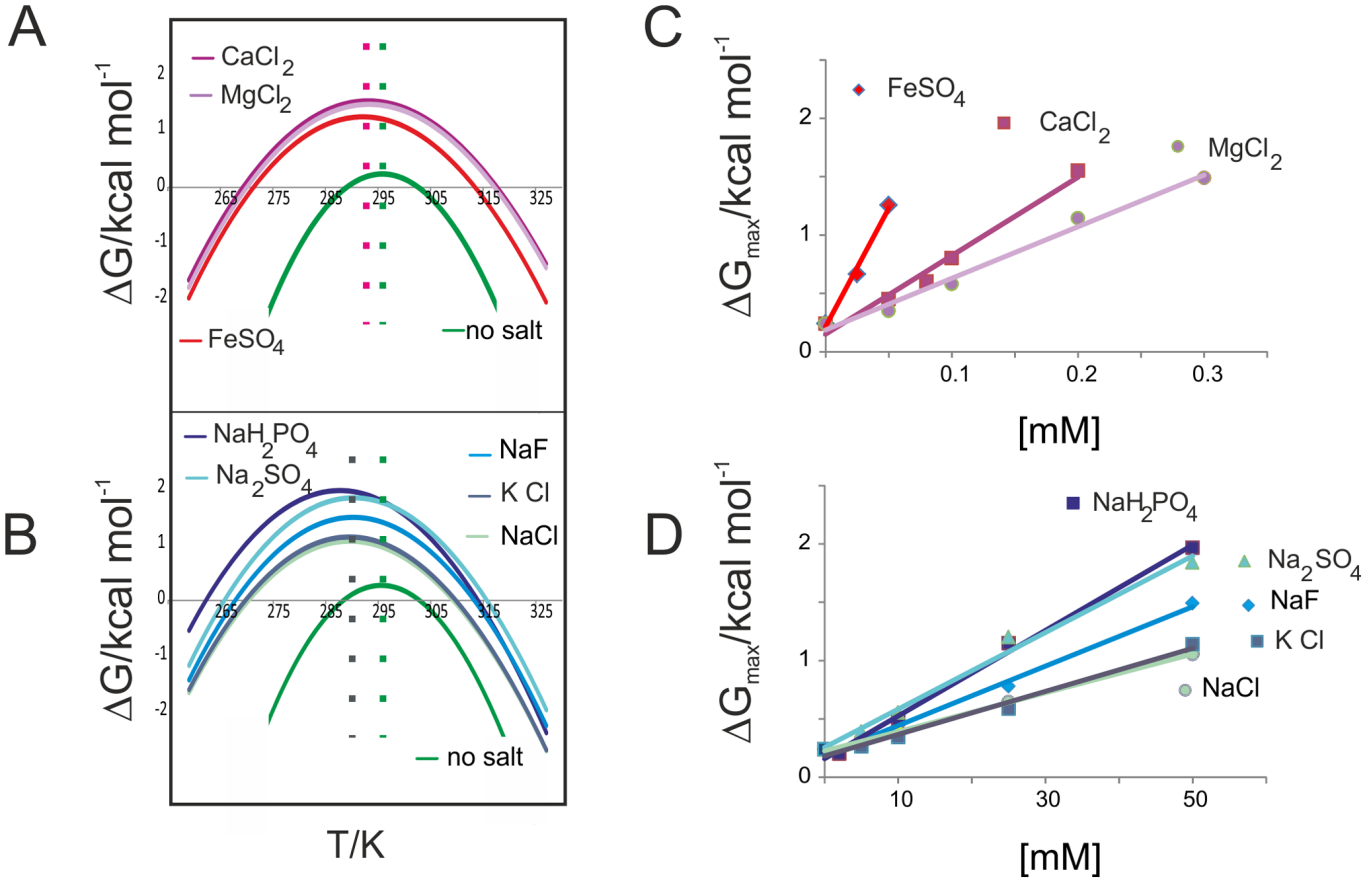
Stability curves and thermodynamic parameters. A) Comparison of the stability curves for Yfh1 in HEPES alone (no salt) and in the presence of three representative salts of divalent cations at concentrations that yield the maximum value of ΔG. The dotted lines show average values of *T_S_*. B) Comparison of the stability curves for Yfh1 in HEPES alone (no salt) and in the presence of five representative salts of monovalent cations at concentrations that yield the maximum value of ΔG. The dotted lines show average values of *T_S_*. C) Dependence of Δ*G_max_* for divalent cations as a function of concentration. D) Δ*G_max_* for all anions as a function of concentration.

The intrinsic difference between divalent cations and all the other ions can be seen immediately from a look at the stability curves for concentrations at which the protein attains almost complete refolding (Figure 3 A,B): typically complete folding requires 50 mM for anions (and their monovalent cations) but 0.3 mM, 0.2 mM and 0.05 mM for Mg^2+^, Ca^2+^ and Fe^2+^ respectively. However, the “low” concentrations of the divalent cations correspond to salt: protein molar ratios of 30, 20 and 5 respectively. These data confirm not only the uniqueness of ferrous ion but also point once again to the fact that the mechanism of interaction of divalent ions is not a generic ionic strength effect. The different concentration range can be appreciated from Figures 3 C and D, which show the concentration dependence of the free energy differences at the maximum of the stability curves. In addition, it is possible to see that the stability curves for the two classes also cluster around different values of *T_S_*, the temperature of maximum stability, with an average value of ca. 20°C for divalent anions and of ca. 17°C for the other ions.

An obvious consequence of the drift of the values of *T_s_* in the presence of salts is that the apparent influence on *T_m_* and *T_c_* values is different, even if the curves are symmetrical. In other words, by looking only at the numerical values of *T_m_* and *T_c_* (see Table 2), one would conclude that cold denaturation is more affected by the value of the ionic strength than is heat denaturation, whereas the relevant physical event is the shift of the whole curve towards lower temperatures. This observation strengthens once more the importance of assessing thermal stability from the whole stability curve rather than from changes in single values (*T_m_* and *T_c_*). The values of *T_S_*, alongside the changes in *T_m_* and *T_c_* compared to buffer alone are summarized in Table 2.

**Table 2:** T_m_ and T_max_ in the presence of selected salts.

HEPES salt/mM	% folded	*ΔT_c_*/°C	*ΔT_m_*/°C	*T_S_*/°C
**HEPES**				
	60	–	–	22.8
**NaF**				
50	93	−21.3	10.6	17.1
**NaCl**				
50	86	−18.2	6.9	17.0
**NaH_2_PO_4_**				
50	97	−26.6	11.3	15.0
**Na_2_SO_4_**				
50	96	−23.1	13.0	17.4
**KCl**				
50	88	−19.0	7.1	16.6
**MgCl_2_**				
0.3	93	−18.9	14.5	20.2
**CaCl_2_**				
0.2	93	−20.4	14.8	20.1
**FeSO_4_**				
0.05	90	−17.5	10.8	19.1

### Mapping the effect onto the protein surface

To check whether the influence of cations is consistent with the electrostatic potential of the surface of the protein, we calculated the electrostatic potential of Yfh1 at zero ionic strength (corresponding approximately to the HEPES buffer) and at 500 mM NaCl. The calculation of the electrostatic potential, based on the well-known method of Nicholls and Honig [17], was done directly within the graphics software (MOLMOL [18]).

**Figure 4:**
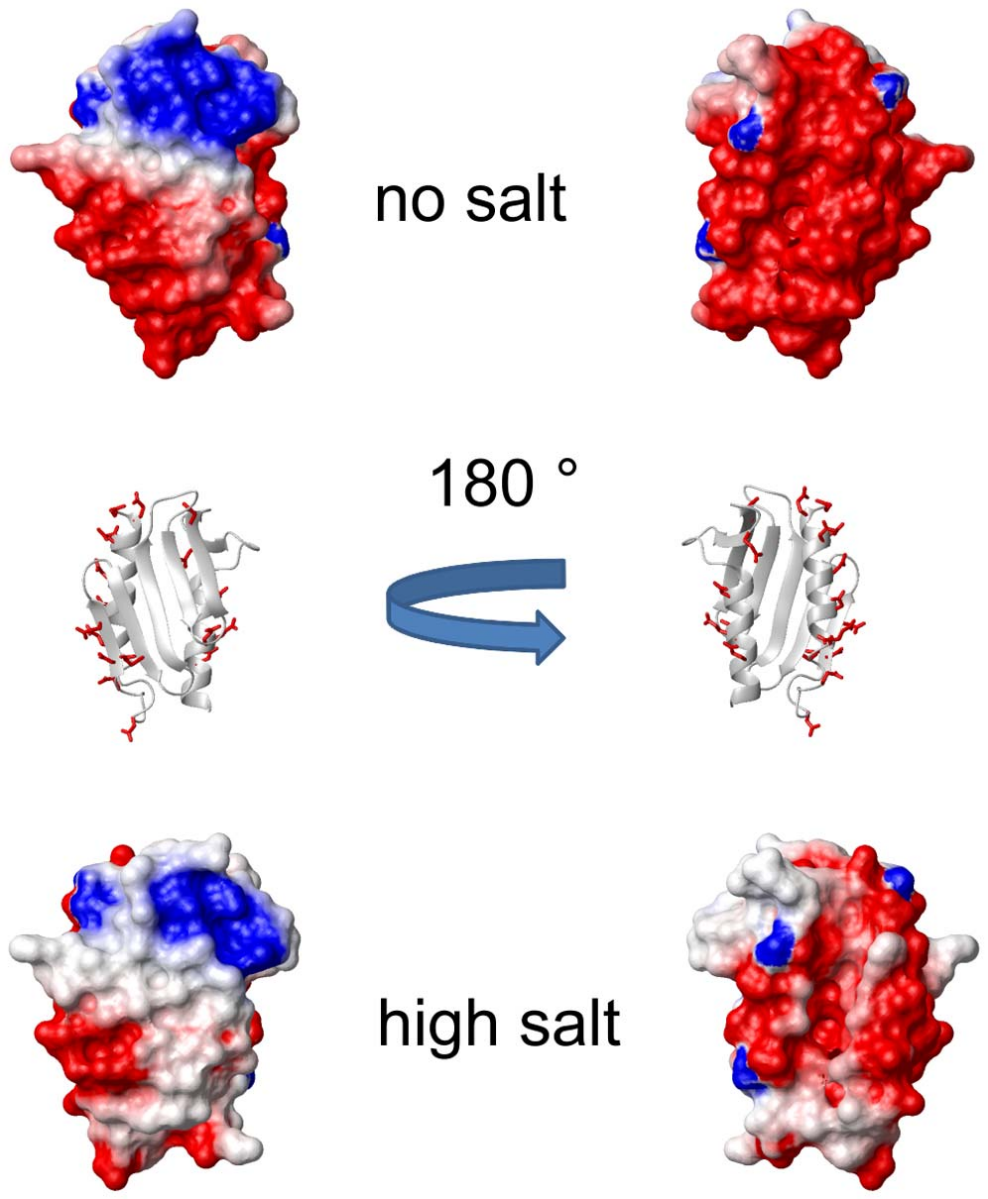
Electrostatic potential of the surface of Yfh1 at low and high ionic strength. The two views of the surface are rotated by 180 ° along the vertical axis, as shown by the ribbon representation models in the middle. The surface on the right shows the ridge α1α1 where most acidic residues are located: the side-chains of acidic residues are shown in red. Molecular models were generated by MOLMOL [18].

**Figure 5:**
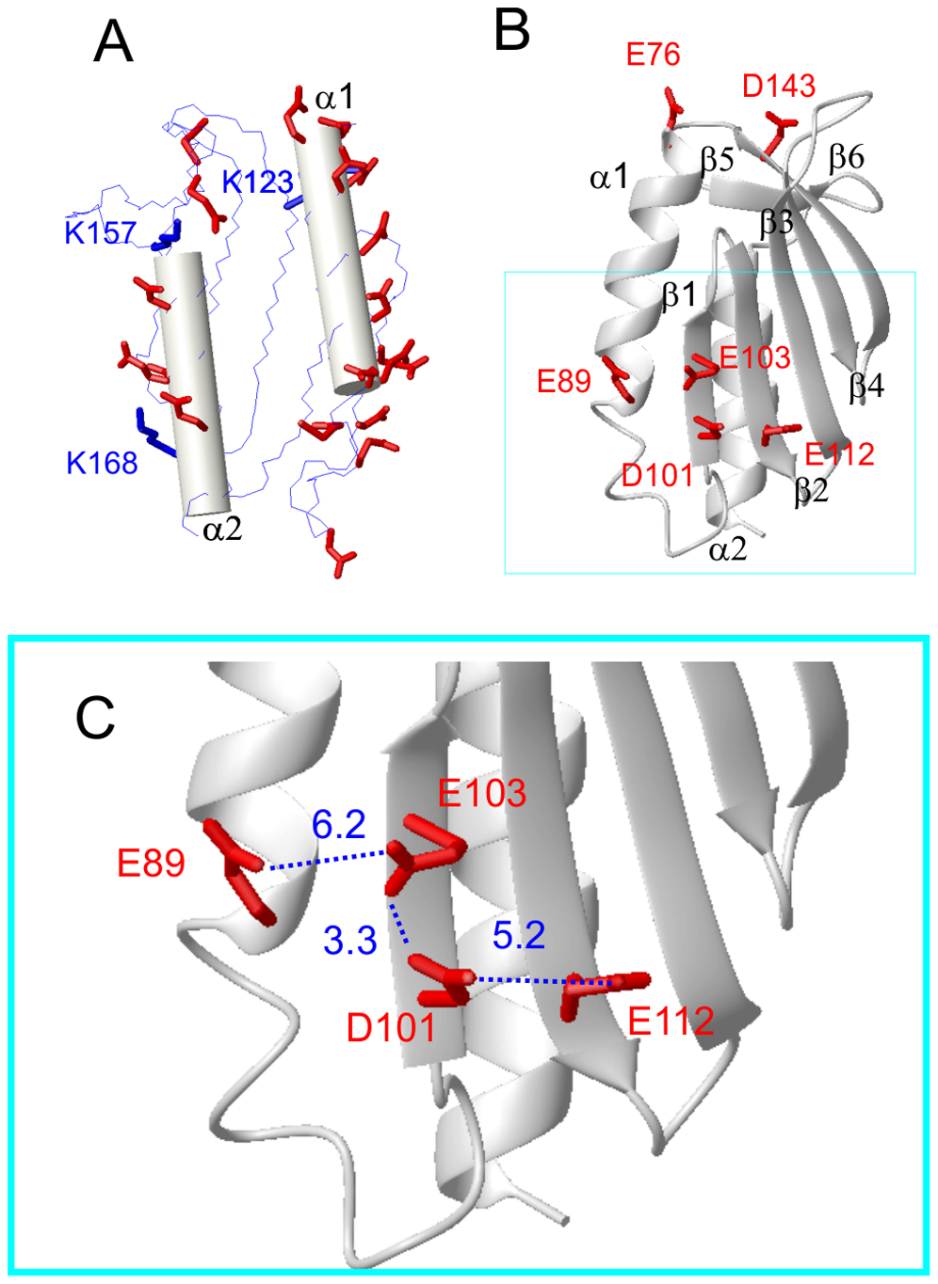
Distribution of acidic residues on the surface of Yfh1. A) Yfh1 surface seen from the side of the two helices (represented as cylinders). The side-chains of *all* acidic residues are shown as red thick sticks, the three basic residues closest to the helices are shown as blue thick sticks. B) Different view of a ribbon representation showing secondary structure elements containing spatially close acidic residues. C) Expanded view of the main electrostatic hinge. Distances of closest contact between oxygen atoms of adjacent residues are highlighted by dotted lines. Residue numbers are those of PDB ID:2FQL. Molecular models were generated by MOLMOL [18].

A comparison of the surface at these two limiting conditions is shown in Figure 4 for two orientations of the protein. Consistent with the pI of the protein (4.2) and its ability to interact with iron ions, the surface appears to be predominantly negatively charged at low salt. At high salt the effect is reduced, consistent with the partial neutralization induced by high concentrations of monovalent cations. However, even the high ionic strength simulation cannot account for a stabilization comparable with very low concentrations of divalent cations (see Table 1). We therefore analyzed the possible structural effects of more specific ion binding by examining the distribution of negatively charged residues in the structure of Yfh1. To do so we used the solid state structure of Yfh1 (PDB ID: 2FQL) which is at much higher resolution than the corresponding NMR structure (PDB ID: 2GA5). As can be seen in Figure 5A, nearly all the acidic residues are located on the two helices. Most of these negatively charged residues are “frustrated”, being uncompensated by adjacent positively charged residues. Figure 5 A shows that K157 and K168 might partially neutralize adjacent negative charges on helix α2, whereas only K123, belonging to β3, is sufficiently close in space to the first negatively charged residues of helix α1. All other basic residues are far from acidic residues. The high concentration of negatively charged residues on the two helices must destabilize them; conversely, it is conceivable that the shielding afforded by external cations will stabilize the two helices, even in the case of non-specific monovalent ions.

A direct consequence of the increase in the ionic strength is a marked stiffening of all helices, as illustrated by the substantial increase of the CD intensity at 220 nm. However, NMR data (*vide infra*) point to a global stabilization of the protein architecture, as suggested by the uniform disappearance of cross peaks for the unfolded forms. Accordingly, it is difficult to rationalise the far greater efficacy of divalent cations on the basis of the global reduction of the surface negative charge, particularly if one takes into account the fact that there is a three order of magnitude concentration difference between the most effective divalent ion and monovalent cations. We hypothesize that the effect of divalent cations is probably linked to their ability to join adjacent acidic residues in a complex salt bridge network involving some crucial residues pinpointed previously [4], [6] but also some new ones. Such an effect can in principle be even more pronounced if the divalent cations can help joining acidic residues belonging to different elements of secondary structure. Figure 5 B shows three potential residue pairs: E76/D143 connecting α1 and β5, E89/E103 connecting α1 and β and D101/E112 connecting β1 and β2. Figure 5 C shows an expanded view of the region containing the main interactions. The three distances (in Å) refer to the closest oxygen atoms between adjacent acidic residues. The whole region may be defined as an “electrostatic hinge”.

### NMR titrations

An important issue related to the interpretation of the results on the thermal stability of Yfh1 in the presence of different salts is whether the origin of the effects must be sought in generic changes of the interactions of the protein with its surrounding (including changes in water structure induced by salts) or in specific interactions. NMR spectroscopy is ideally suited to address this issue.

**Figure 6:**
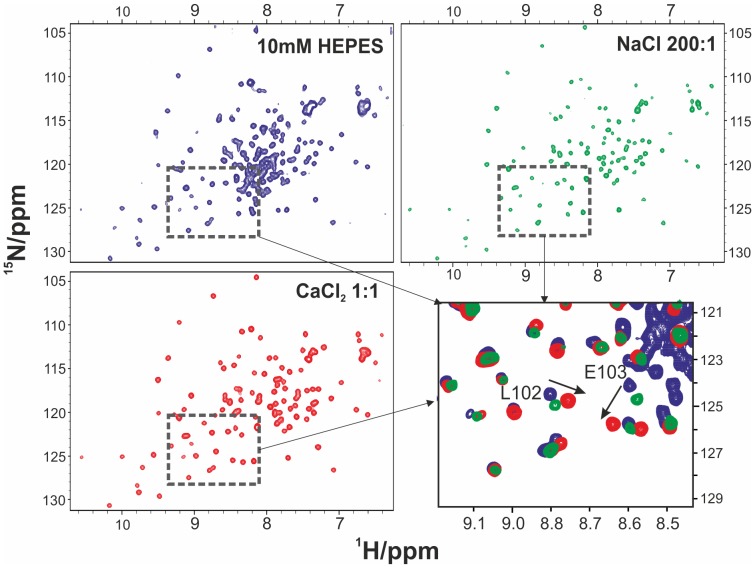
Comparison of NMR spectra of Yfh1. A) ^15^N-HSQC NMR spectra of 0.25 mM Yfh1 in HEPES (blue cross peaks). B) (green cross peaks) in the presence of 200∶1 NaCl. ^15^N-HSQC NMR spectra of 0.25 mM Yfh1 in HEPES and C) (red cross peaks) in the presence of 1∶1 CaCl_2_. The fourth panel (D, lower right) shows the superposition of the region of the three spectra in which it is possible to observe the largest changes. The outstanding chemical shift changes involving L102 and E103 are highlighted by arrows Residue numbers are those of PDB ID:2FQL.

Figure 6 shows a comparison of the spectra of Yfh1 in samples containing a concentration of monovalent cation in a molar ratio of 200∶1 (ion: protein) and a concentration of divalent cation in a 1∶1 molar ratio respectively. When recorded in the presence of salt concentrations comparable to that of the protein^ 15^N-^1^H HSQC spectra are indistinguishable from those recorded in HEPES if the salt contains a monovalent cation (NaCl, data not shown) whereas they are profoundly and specifically affected in the presence of a divalent cation (CaCl_2_, Figure 6C). Both spectra of Figure 6B and of Figure 6C show a complete disappearance of cross peaks (particularly the cluster around 8 ppm and 120 ppm for protons and nitrogens respectively) of the unfolded species. However, those of the folded species are very close to those in HEPES for the NaCl solution whereas specific displacements can be observed for several peaks of the folded forms in the presence of CaCl_2_ (see Figure 6D). It is reassuring that the two outstanding chemical shift changes observed in the HSQC in the presence of Ca^2+^ are those of E103 and of L102, consistent with the hypothesis of a specific interaction in this region (Figure 5C).

## Discussion

Understanding the factors that stabilize proteins is essential for rationalizing the forces that determine protein folding and for understanding their function. Frataxins provide an interesting example, as they form a family of highly conserved essential proteins. The availability of sequences from different organisms, both mesophiles and extremophiles, provides a great opportunity for comparative studies. Preliminary analysis has shown that the thermodynamic stability of different frataxin orthologs vary considerably and, as expected, are influenced by quite different mechanisms. Overall, the *E. coli* and human proteins are much more stable than the close orthologs from a psychrophilic organism and from yeast. While these results can easily be correlated with environmental factors for the psychrophilic protein, it is significantly more difficult is to rationalize them for the yeast protein.

We previously demonstrated that a factor that plays an important role in the low stability of the yeast protein is its unusually short C-terminal sequence, which does not provide effective protection of the hydrophobic core [6]. When present, these additional residues form an unstructured (but not flexible) tail that inserts between two helices and shields the hydrophobic core. In support of this hypothesis is the evidence that elongation of Yfh1 to the length of the human sequence stabilizes the protein, while shortening human frataxin to the length of the yeast protein leads to a significant reduction in stability. Another mechanism that could play a role in the lower stability of the psychrophilic frataxin is the presence of two histidines, H44 and H67, not conserved in other frataxins, that could contribute to stabilize the fold and reduce the flexibility of the protein through formation of hydrogen bonds with negatively charged partners (E42, D27 and D78) only at basic pH [7]. Finally, a further interesting stabilizing factor is that frataxins are very sensitive to the presence of salts [5], [6]. Here, we have carried out the first detailed analysis of the effect of ionic strength on the stability of the yeast ortholog as a function of temperature. A difficulty in this study was that of discriminating between specific binding and the effect of bulk ion shielding. We were aided in solving this problem by the properties of Yfh1. In addition to being less stable than the other members of its family and a protein with an extreme sensitivity to salts, this protein has the unusual property of undergoing cold denaturation at accessible temperatures when at low ionic strengths. This behavior provided us with a unique and robust tool to systematically measure the whole stability curve, which is much more informative than measurement of the high temperature melting point [9]. As anticipated by our preliminary studies [5], [6], [19] protein stability is enhanced by all ions investigated. Cations are much more effective than anions, to the point that the latter do not seem to play a significant role in stabilization, apart from counterbalancing their cations. Salt effects on protein stability are not an absolute novelty, but previous studies [20], [21] have generally focused on the effect of very high concentrations (up to 6 M) typical of extremophiles. In the present study we have found a crucial region in the structure of Yfh1 where the proximity of negatively charged residues can be described as a potential “electrostatic hinge”: the network of E89, E103, D101 and E112, illustrated in Figure 5, can be highly destabilizing if uncompensated by positive charges, as supplied by even very low concentrations of divalent cations. It is significant that this network is not fully conserved in the *E. coli* and human frataxins that are more stable than yeast frataxins. The comparison between the corresponding regions of these three proteins is shown in Figure S3. In the case of the bacterial ortholog (Cyay) the netwok is reduced to three residues, namely the partially conserved E18 and E33 and the conserved D31, whereas the human frataxin in the same region has only E111 and D124 joining helix α1 and β1. In addition, the residues located in different secondary structure elements are further apart than those in Yfh1.

Our analysis might have consequences for our molecular understanding of cold denaturation and of the effects that allow this to occur for Yfh1 but not for Cyay and human frataxin. The mutual repulsion of residues of the hinge keeps different secondary structure elements separated, thus facilitating the access of the solvent to the hydrophobic core, in agreement with the mechanism of solvation of core residues proposed by Privalov to explain the mechanism of cold denaturation [22]. A similar situation could thus be used to explain the behavior of other proteins. The bacterial protein IscU, which, like frataxin, participate in the iron-sulfur cluster assembly machinery, has also been shown to undergo cold denaturation above the freezing point of water. Interestingly, IscU is also a highly negatively charged protein (pI around 4.5) with large clusters of uncompensated glutamates and aspartates. These results could inspire the identification of new examples of proteins undergoing cold denaturation which could then be used to further study this still under-characterized phenomenon.

## Conclusions

We studied the thermal stability of Yfh1, the yeast ortholog of frataxins, in the presence of several salts over a wide concentration range. By exploiting the unique property of Yfh1 to undergo cold denaturation above the freezing point of water, we could measure the full stability curves for all conditions. The data extracted from full stability curves are far more reliable than previously determined stabilities based only on high temperature unfolding points. On the basis of this quantitative evaluation it is possible to disentangle simple ionic strength effects from specific ionic interactions. The surface of Yfh1 is exceptionally rich in negative charges: accordingly, it is understandable that a partial decrease in repulsions between these charges, as induced by substantial increases in the ionic strength, can stabilize the protein. However, the divalent cations are effective at concentrations that are two to three orders of magnitude lower. A possible explanation for this phenomenon resides in the binding of divalent ions at sites characterized by networks of spatially close negative residues that would otherwise destabilize the fold. This finding hints at a possible double role of iron ions, not only as ions involved in the building of iron-sulphur clusters but also as key stabilizers of the protein fold. It may be hypothesized that ferrous ions take part in a kind of switching mechanism: iron-loaded Yfh1 binds more strongly to the other components of the iron-sulphur cluster machinery but when iron is released to form the cluster leave a destabilized protein that can dissociate more easily.

## Supporting Information

Figure S1 **CD spectra of Yfh1 at 0°C, 20°C and 40°C in the presence of different salts.** The concentration of salt is 2 mM in all cases.
(TIF)

Figure S2 **Fitting of the unfolding curves at increasing concentration of KCl.** The green dots are the experimental data, the black line is the fitting curve and the blue line is representing the agreement between the experimental data and the fitting curve.
(TIF)

Figure S3 **Close up view of the main electrostatic hinge in thre frataxins.** Yfh1 residue numbers are those of 2FQL pdb, Cyay numbers are those of 1EW4 pdb, hfrat numbers are those of 1EKG pdb. Distances of closest contact between oxygen atoms of adjacent residues are highlighted by dotted lines.Molecular models were generated by MOLMOL.
(TIF)

Text S1 **Extraction of thermodynamic parameters from data.**
(DOC)
