# Supplementary figures and images for: Yeast Frataxin Is Stabilized by Low Salt Concentrations: Cold Denaturation Disentangles Ionic Strength Effects from Specific Interactions
Source: PLoS One. 2014 May 6;9(5):e95801. doi: 10.1371/journal.pone.0095801 (PMC4011691; doi:10.1371/journal.pone.0095801)

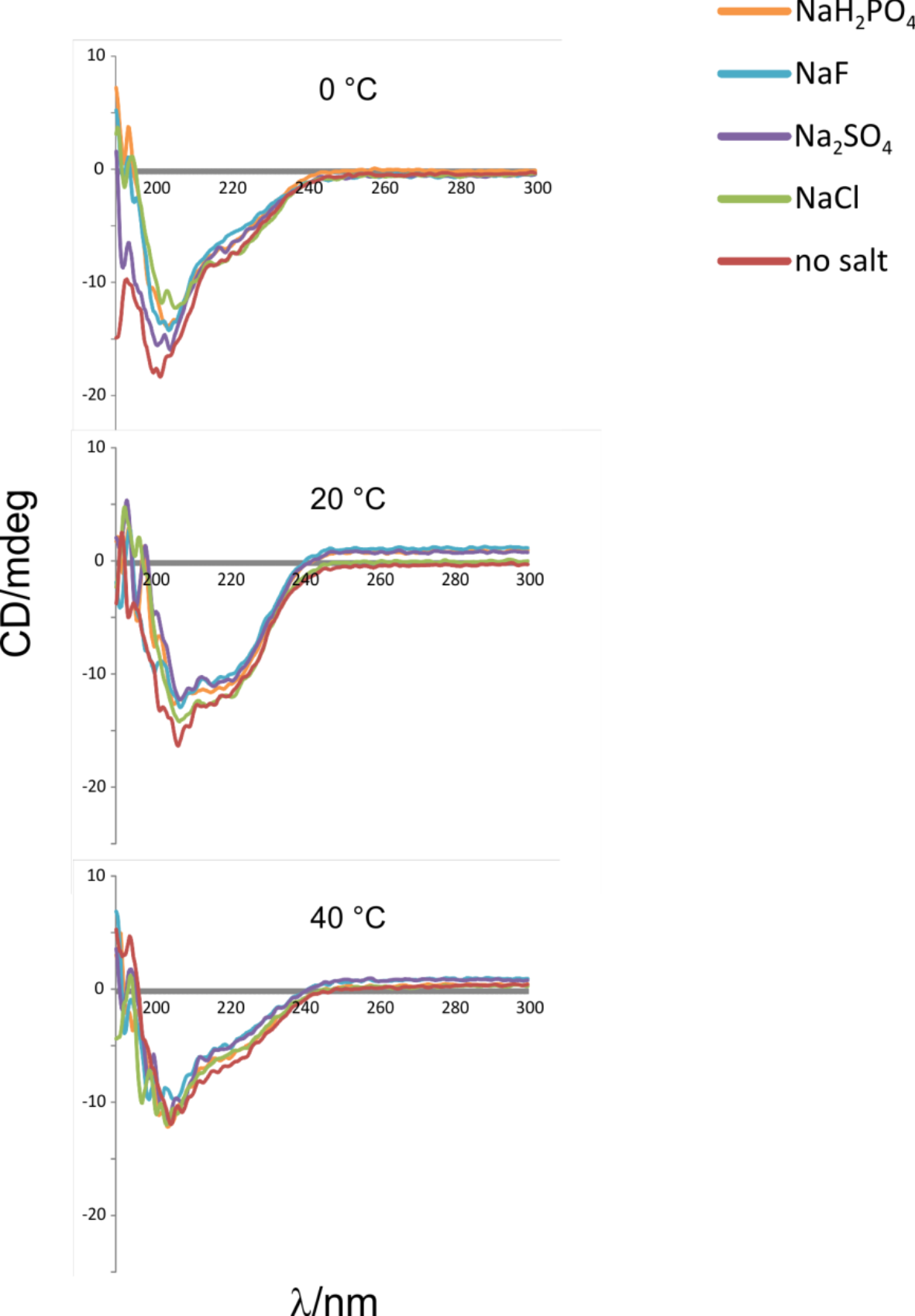

Supplement: Figure S1 — CD spectra of Yfh1 at 0°C, 20°C and 40°C in the presence of different salts. The concentration of salt is 2 mM in all cases. (TIF) [file pone.0095801.s001.tif]

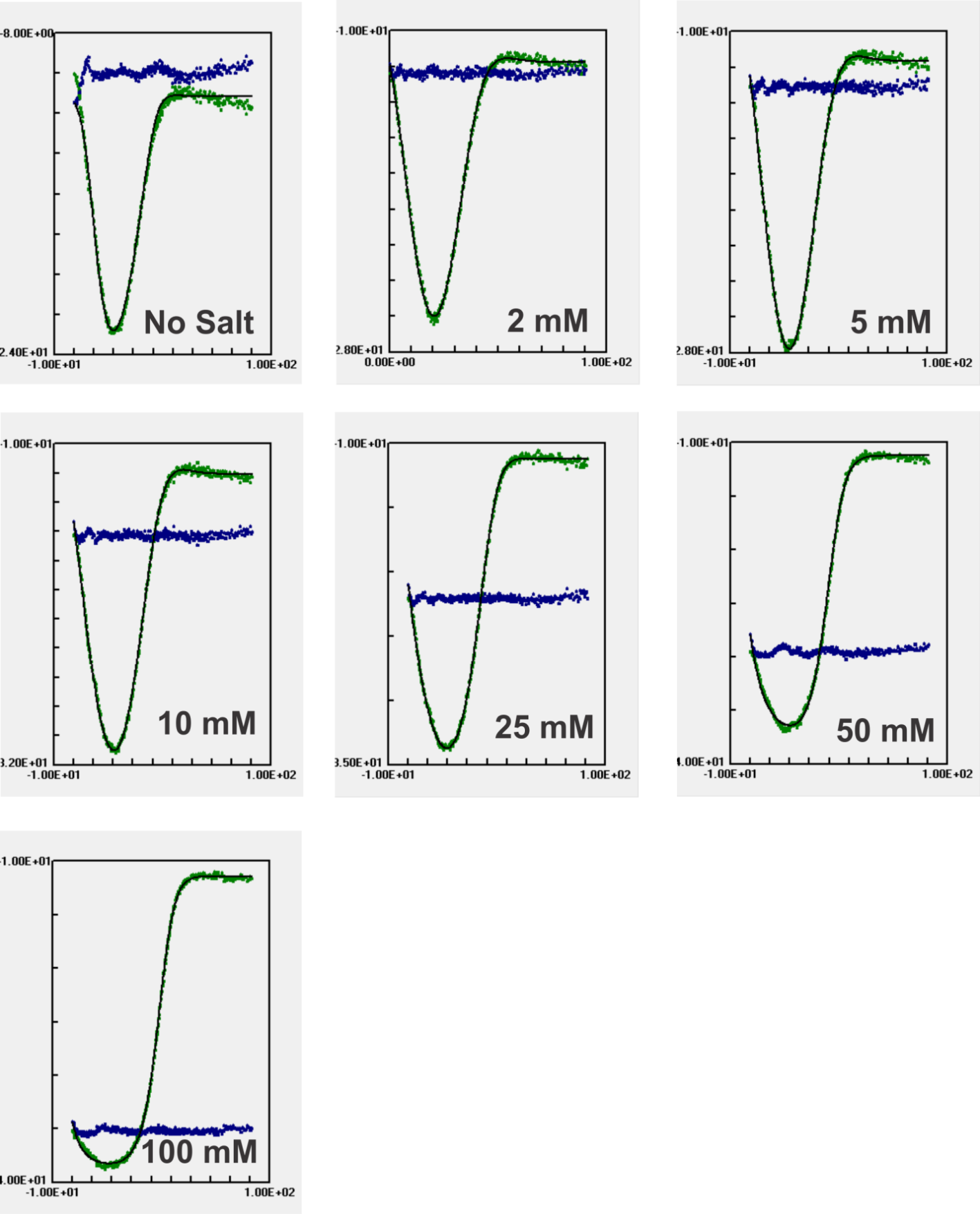

Supplement: Figure S2 — Fitting of the unfolding curves at increasing concentration of KCl. The green dots are the experimental data, the black line is the fitting curve and the blue line is representing the agreement between the experimental data and the fitting curve. (TIF) [file pone.0095801.s002.tif]

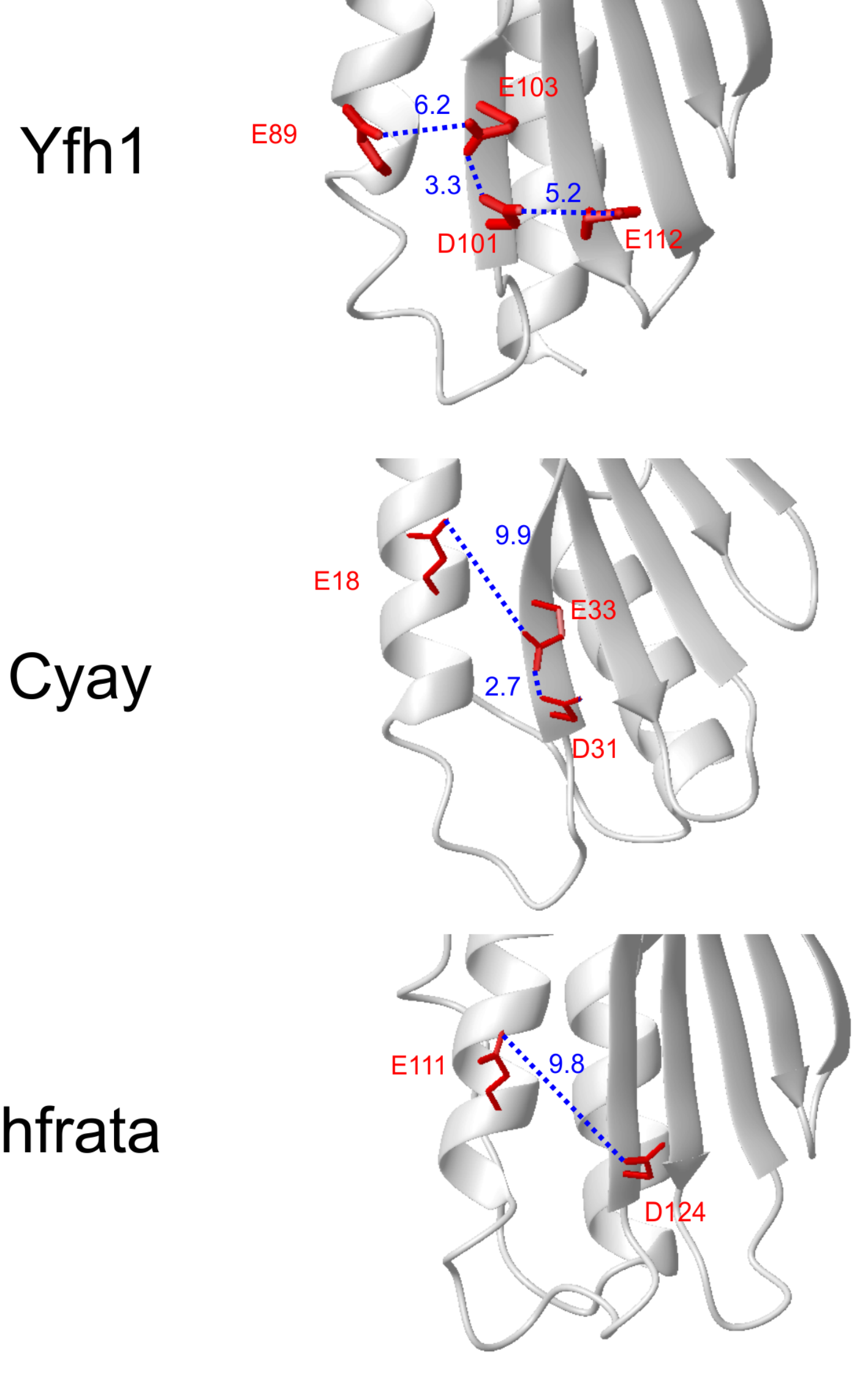

Supplement: Figure S3 — Close up view of the main electrostatic hinge in thre frataxins. Yfh1 residue numbers are those of 2FQL pdb, Cyay numbers are those of 1EW4 pdb, hfrat numbers are those of 1EKG pdb. Distances of closest contact between oxygen atoms of adjacent residues are highlighted by dotted lines.Molecular models were generated by MOLMOL. (TIF) [file pone.0095801.s003.tif]
