# Supplementary material for: Yeast Frataxin Is Stabilized by Low Salt Concentrations: Cold Denaturation Disentangles Ionic Strength Effects from Specific Interactions
Source: PLoS One. 2014 May 6;9(5):e95801. doi: 10.1371/journal.pone.0095801 (PMC4011691; doi:10.1371/journal.pone.0095801)
Supplement: Text S1 — Extraction of thermodynamic parameters from data. (DOC) [file pone.0095801.s004.doc]

Thermal Unfolding

Analysis of CD data: extraction of thermodynamic parameters.

Signal measured during thermal unfolding (S_OBS_) can be related to the fraction of folded protein (F_f_):

S_OBS_ = S_f_F_f_ + S_u_F_u_ (1)

Substituting F_f_ = 1- F_u_ in equation (1) gives:

S_OBS_ = S_f_(1-F_u_) + S_u_F_u_

F_u_ can be related to the K_u_ (unfolding constant) using the following equation:

F_u_ = K_u_/(1+K_u_) (2)

S_OBS_ = S_f_
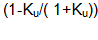
 *+* S_u_
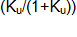


S_OBS_ =
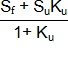


Knowing that ΔG = -RTLn K_u_ we obtain:

S_OBS_ =
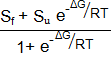


Finally using the Gibbs Helmholtz equation:

ΔG_T_ = ΔH_Tm_ [1-(T/T_m_)] + ΔC_p_ {(T-T_m_) – TLn (T/T_m_)} (3)

We substitute the ΔG and obtain the final equation that fits the CD signal as following:

S_OBS_ =
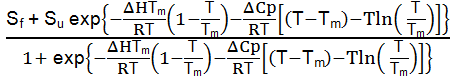
 (4)

Equation (4) allows the measurements of T_m_, ΔH_Tm_ and ΔC_p_ from the CD curve: the data are then used to calculate back the ΔG of unfolding using the modified Gibbs Helmholtz equation. Plots A and B in Figure 4, of the main paper are built in this way: plots A and B help also for the determination of T_c_ (temperature of cold denaturation) which is one of the two temperatures where ΔG=0.

For our analysis of thermal unfolding we used in house developed software from M.S. which tries to fit the unfolding curves as best as possible. The software provided the thermodynamic parameter based on equation (4). Figure S2P, shows the fitting of the curves at increasing salt concentration.

Determination of Folded Fraction

The calculation of the folded fraction at the temperature of maximum stability is obtained from the following equation:

ΔG = −RTLn K_u_ = −RTLn [F_u_/(1-F_u_)] = −RTLn (F_u_/F_f_)
